# Early therapeutic persistence on dabigatran versus warfarin therapy in patients with atrial fibrillation: results from the Outcomes Registry for Better Informed Treatment of Atrial Fibrillation (ORBIT-AF) registry

**DOI:** 10.1007/s11239-018-1715-1

**Published:** 2018-07-26

**Authors:** Larry R. Jackson, Sunghee Kim, Peter Shrader, Rosalia Blanco, Laine Thomas, Michael D. Ezekowitz, Jack Ansell, Gregg C. Fonarow, Bernard J. Gersh, Alan S. Go, Peter R. Kowey, Kenneth W. Mahaffey, Elaine M. Hylek, Eric D. Peterson, Jonathan P. Piccini

**Affiliations:** 1 Duke Clinical Research Institute, Duke University Medical Center, Durham, NC USA; 2 Hofstra North Shore/LIJ School of Medicine, Hempstead, New York, USA; 3 UCLA Division of Cardiology, Los Angeles, CA USA; 4 Mayo Clinic College of Medicine, Rochester, MN USA; 5 Kaiser Permanente, Oakland, CA USA; 6 Lankenau Institute for Medical Research, Jefferson Medical College, Wynnewood, PA USA; 7 Division of Cardiovascular Medicine, Stanford Center for Clinical Research, Stanford University School of Medicine, Stanford, CA USA; 8 Boston University School of Medicine, Boston, MA USA; 9 Division of Cardiovascular Medicine, Duke Clinical Research Institute, Duke University Medical Center, 2400 Pratt Street, Suite 7009, Durham, NC 27705 USA

**Keywords:** Atrial fibrillation, Oral anticoagulation, Dabigatran, Warfarin

## Abstract

Anticoagulation is highly effective for the prevention of stroke in patients with atrial fibrillation (AF) but it is dependent on patients continuing therapy. While studies have demonstrated suboptimal therapeutic persistence on warfarin, few have studied persistence rates with non vitamin K antagonist oral anticoagulants (NOACs) such as dabigatran. We examined rates of continued use of dabigatran versus warfarin over 1 year among AF patients in the ORBIT-AF registry between June 29, 2010 and August 09, 2011. Multivariable logistic regression analysis was used to identify characteristics associated with 1-year persistent use of dabigatran therapy or warfarin. At baseline, 6.4 and 93.6% of 7150 AF patients were on dabigatran and warfarin, respectively. At 12 months, dabigatran-treated patients were less likely to have continued their therapy than warfarin-treated patients [Adjusted persistence rates: 66% (95% CI 60–72) vs. 82% (95% CI 80–84), *p* < .0001]. Predictors of dabigatran persistence included: CHA_2_DS_2_-VASc risk scores ≥ 2 OR 5.69, (95% CI 1.50–21.6) and BMI greater than 25 mg/m^2^ but less than 38 kg/m^2^ 1.05 (1.01–1.09). Predictors of persistence on warfarin included: African American race (vs. White) 1.53 (1.07–2.19), Hispanic ethnicity (vs. White) 1.66 (1.06–2.60), paroxysmal and persistent AF (vs. new-onset) 1.68 (1.21–2.33) and 1.91 (1.35–2.69) respectively, LVH 1.40 (1.08–1.81), and CHA_2_DS_2_-VASc risk scores ≥ 2 1.94 (1.18–3.19). While 1-year persistence rates for dabigatran were lower than warfarin, persistence rates for both agents were not ideal. Future studies evaluating contemporary persistence are needed in order to assist in better targeting interventions aimed to improve anticoagulation persistence.

**Electronic supplementary material:**

The online version of this article (10.1007/s11239-018-1715-1) contains supplementary material, which is available to authorized users.

## Highlights

- There is a paucity of real-world data on the persistence of drug therapy between warfarin and non vitamin K antagonist oral anticoagulants
- ORBIT-AF was used to compare the persistence of warfarin vs. dabigatran in a contemporary cohort of AF patients
- Warfarin persistence was greater than dabigatran at 6 and 12 months, respectively
- Future studies evaluating persistence of other non vitamin K antagonist oral anticoagulants as well as the implementation of effective strategies to improve persistence are needed

## Introduction

Guideline recommended management of patients with AF includes long-term anticoagulant prophylaxis to prevent ischemic stroke in patients with more than 1 risk factor for stroke [1]. Traditional vitamin K antagonist (VKA) oral anticoagulants such as warfarin were previously the gold standard for stroke prevention but require dose adjustments, frequent coagulation laboratory monitoring, vigilance over numerous potential drug–drug interactions, and increased risk of bleeding; all factors that can potentially lead to drug discontinuation. Direct oral anticoagulants such as dabigatran are currently being used for the prevention of stroke and systemic embolism in patients with non-valvular AF [2]. However, it is unclear whether persistence with dabigatran exceeds that of warfarin.

Accordingly, we used the Outcomes Registry for Better Informed Treatment of Atrial Fibrillation (ORBIT-AF) to perform the following: (1) compare the persistence of dabigatran early after the introduction in clinical practice versus warfarin therapy (2) examine predictors associated with the persistence of each drug; and (3) describe the stated indications for discontinuation of each drug.

## Methods

### Study population

We used the ORBIT-AF registry to assess persistence rates for dabigatran and warfarin over 1-year of follow-up. Between June 29, 2010 and August 09, 2011, 7150 patients treated with warfarin [N = 6691(93.6%)] and dabigatran [N = 459 (6.4%)] at baseline were enrolled in the ORBIT-AF registry. The rationale and design of the ORBIT-AF registry have been previously described [3].

### Data collection and study endpoints

Persistence with dabigatran and warfarin were defined as continuous use between baseline, 6 months, and 1-year follow-up. If a patient discontinued taking dabigatran or warfarin at 6 months or 1 year, for any reason, he or she was defined as discontinuing dabigatran or warfarin and therefore, not persistent. For those patients who discontinued either dabigatran or warfarin at their 6 months-and/or 1-year follow-up, providers were asked to identify one or more primary and secondary reasons for discontinuation from a pre-specified list [4].

### Statistical analysis

We compared baseline characteristics between patients treated with dabigatran and patients treated with warfarin over 1 year of follow-up. The differences across two groups were assessed using Wilcoxon rank-sum test for continuous variables and the chi square test for categorical variables. The data are presented as medians (interquartile range) for continuous variables and as percentages for categorical variables. In order to assess the difference of persistence rates between warfarin and dabigatran at 6 months or 1 year, a *p* value will be presented using chi square test. Adjusted persistence rates were calculated using inverse probability weighting [IPW] analysis incorporating propensity scores to minimize difference between people taking dabigatran and warfarin. The propensity score was obtained from a logistic regression model for dabigatran use [4]. Persistence rates for both warfarin and dabigatran were then re-calculated using inverse propensity weighting to balance the characteristics of patients receiving these two treatments. In addition, 6 and 12 month adjusted persistence rates were calculated among specific subgroups including: age > 75, women, creatinine clearance < 50 ml/min/1.73 m^2^, and concomitant antiplatelet therapy. Multivariable logistic regression was used to determine factors associated with persistence of dabigatran and warfarin. Local institutional review boards approved this study.

## Results

### Patient characteristics

Patients treated with dabigatran were younger (median 71 vs. 74 years, *p* < .0001), had higher left ventricular ejection fractions, higher creatinine clearance (88 vs. 77 ml/min/1.73 m^2^, *p* < .0001), lower CHA_2_DS_2_-VASc risk scores, and fewer prior stroke or transient ischemic attacks events (11 vs. 16%, *p* = .003) than those treated with warfarin. Patients treated with dabigatran had more severe symptoms (EHRA class III: 20 vs. 14%, *p* < . 0001), higher rates of management with a rhythm control strategy (43 vs. 28%, *p* < . 0001), more attempts at cardioversion (38 vs. 32%, *p* < . 006), and more frequent catheter ablation of AF (10 vs. 5%, *p* < . 0001*)* than those treated with warfarin.

### Persistence of therapy

**Fig. 1:**
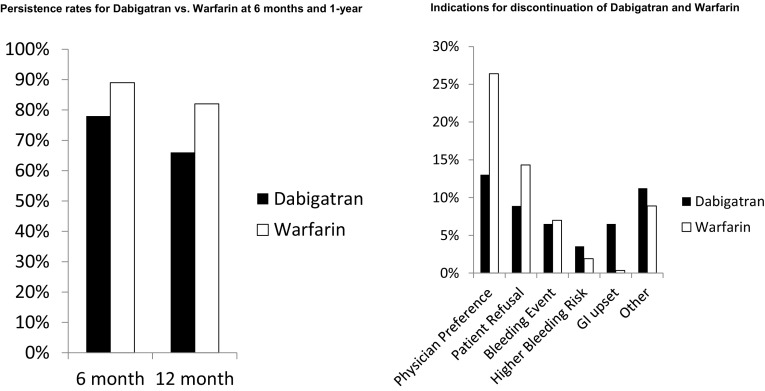
Persistence rates and indications for discontinuation of dabigatran and warfarin

Adjusted 6 and 12 month persistence rates were as follows: 6 months, dabigatran versus warfarin: 78% (95% CI 72–84) versus 89% (95% CI 87–90), *p* < .0001; 12 months, dabigatran versus warfarin: 66% (95% CI 60–72) versus 82% (95% CI 80–84), *p* < . 0001 (Fig. 1). The number of patients who discontinued warfarin and dabigatran at 6 and 12 months is as follows: {6 months: warfarin [675 (10.5%)], dabigatran [104 (23.8%)]; 12 months: warfarin [1159 (17.3%)], dabigatran [169 (36.8%)]}. We analyzed persistence of therapy in several special patient groups including: age > 75, females, estimated creatinine clearance (CrCl) less than 50 ml/min/1.73 m^2^ (Cockcroft-Gault), and patients receiving concomitant antiplatelet therapy (ASA or P2Y_12_ receptor inhibitor). All groups demonstrated lower adjusted 6 month and 1 year persistence for dabigatran versus warfarin (Supplemental Material).

### Characteristics and predictors of warfarin and dabigatran persistence

Predictors of persistence of warfarin included: age, duration of AF < 3 years, African American race and Hispanic ethnicity, LVH, and more permanent forms of AF such as persistent and permanent AF. Predictors of persistent use of dabigatran included: medium and high CHA_2_DS_2_-VASc scores, defined as a score of 1 and ≥ 2 respectively, and BMI greater than 25 but less than or equal to 38 kg/m^2^ (Table 1).

**Table 1:** Predictors of dabigatran and warfarin persistence

Risk factor	Dabigatran adjusted HR (95% CI)	*p* value	Warfarin adjusted HR (95% CI)	*p* value
Duration of AF < 3 years	–	–	1.15 (1.10–1.22)	<. 0001
Prior catheter ablation of AF	–	–	0.58 (0.45–0.74)	<. 0001
Most recent ECG-sinus rhythm	–	–	0.72 (0.61–0.85)	<. 0001
Age, years (per 10 year increase)	–	–	1.14 (1.06–1.22)	.0002
Persistent AF vs. first onset	–	–	1.91 (1.35–2.69)	.0002
Cognitive impairment	–	–	0.56 (0.40–0.80)	.001
Paroxysmal AF vs. first onset	–	–	1.68 (1.21–2.33)	.002
Heart rate > 80	–	–	0.94 (0.90–0.98)	.004
CHA_2_DS_2_VASC high vs. low	5.69 (1.50–21.55)	.01	1.94 (1.18–3.19)	.009
25 < BMI ≤ 38	1.05 (1.01–1.09)	.02	–	
LVH	–	–	1.40 (1.08–1.81)	.01
Permanent AF vs. first onset	–	–	1.55 (1.08–2.23)	.02
African American vs. White	–	––	1.53 (1.07–2.19)	.02
Hispanic vs. White	–	–	1.66 (1.06–2.60)	.03
CHA_2_DS_2_VASC medium vs. low	3.95 (0.95–16.38)	.06	1.12 (0.66–1.92)	.67

### Indications for discontinuation

The most commonly reported reasons for dabigatran discontinuation were physician preference, other indications, and patient refusal followed by bleeding events, GI upset, and high bleeding risk (Fig. 1). Similarly, the two most common reasons for warfarin discontinuation were physician preference and patient refusal.

## Discussion

Quality care to reduce the risk of stroke in patients with AF requires both the initiation of stroke prevention and persistent therapy over the long-term. Our analysis yields several important findings. First and foremost, 1-year persistence rates for patients who received warfarin were higher than those receiving dabigatran. Patients who persisted on warfarin were older, and more likely to be an underrepresented minority, increased number of co-morbid medical illness, and to have more permanent forms of AF compared to patients who persisted on dabigatran. Finally, the most frequent reasons for discontinuation for both warfarin and dabigatran are physician preference, patient refusal, and bleeding.

For decades, warfarin has been the standard of care oral anticoagulant with respect to the prevention of stroke and systemic embolism for patients with AF [5, 6]. While dabigatran is an attractive alternative to warfarin with significant benefits, it is not clear which agent has better persistence with therapy over time. In the RE-LY trial, Connolly et al. showed that 2-year persistence rates were higher for warfarin compared with dabigatran (83 vs. 79%) [2]. Alternatively, in a retrospective cohort using administrative claims data, Zalesak et al., reported that patients who initiated dabigatran treatment demonstrated higher persistence rates than those receiving warfarin therapy at both 6 months (72 vs. 53%) and 1-year (63 vs. 39%) [7].

Our results are from a prospective, contemporary cohort of AF patients. We report a higher rate of warfarin persistence compared to dabigatran, and significantly higher rates of warfarin persistence than prior studies [8–10]. The higher rates of persistence in this study may reflect contemporary trends of enhanced utilization of resources used to monitor and manage warfarin therapy or participation by patients in a registry focused on oral anticoagulation and quality of care. Of note, in the multivariable models for persistence, prior warfarin therapy was not a significant predictor of continued warfarin or dabigatran persistence at 1-year. In addition, warfarin persistence may have been higher due to a longer history of prevalent warfarin use compared to dabigatran, which would promote greater familiarity with warfarin therapy. The lower persistence rates of dabigatran cannot be entirely attributed to actual drug therapy but circumstances between patients taking dabigatran versus warfarin including drug switching or NOAC initiation and not therapeutic failure of drug therapy with dabigatran.

Despite the fact that persistence was higher with warfarin, our data also show that current persistence rates with dabigatran may be higher than those previously reported. For example, an analysis of pharmacy claims data from October 29, 2010 through June 30, 2011 by Tsai et al. found that dabigatran persistence was approximately 60% at 6 months [11] compared with 79% in ORBIT AF. Similar to the GLORIA investigators, our study documents high levels of dabigatran persistence [12].

Physician preference was the primary indication for discontinuation of both warfarin and dabigatran. Previous studies have shown that indications for warfarin discontinuation are primarily guided by physician preference suggesting that long-term persistence with warfarin may be affected by physician concern for safety when prescribing this drug [4, 13]. In addition, our findings confirm work from prior studies showing that bleeding events and gastrointestinal side effects are common reasons for discontinuation of dabigatran [14].

## Limitations

Several limitations need to be acknowledged when considering these data. First, as with all observational analyses, we cannot exclude that after adjustment, the possibility of residual measured or unmeasured confounding exist which may have led to an overestimation and underestimation of warfarin and dabigatran persistence, respectively. Second, the ORBIT-AF study population was derived from practices participating in a US registry and may not be representative of all AF patients in general. In addition, the enrollment of patients for this analysis occurred shortly after the approval of dabigatran (October 2010) and may not represent contemporary persistence trends with this specific direct oral anticoagulant. Finally, we did not analyze the impact of non-persistence on clinical outcomes, although prior studies have demonstrated that persistence with anticoagulation therapy is strongly associated with outcomes [15, 16].

## Conclusion

In this prospective non-randomized observational comparison of ORBIT registry patients with AF who received dabigatran early after introduction into clinical practice versus warfarin, we found significantly lower persistence with dabigatran treated patients within 6 months and 1-year.

## Electronic supplementary material

Below is the link to the electronic supplementary material.

Supplementary material 1 (DOCX 125 KB)
