# Supplementary material for: Early therapeutic persistence on dabigatran versus warfarin therapy in patients with atrial fibrillation: results from the Outcomes Registry for Better Informed Treatment of Atrial Fibrillation (ORBIT-AF) registry
Source: J Thromb Thrombolysis. 2018 Jul 26;46(4):435–9. doi: 10.1007/s11239-018-1715-1 (PMC6182370; doi:10.1007/s11239-018-1715-1)
Supplement: Supplementary file 1 — Supplementary material 1 (DOCX 125 KB) [file 11239_2018_1715_MOESM1_ESM.docx]

**SUPPLEMENTAL MATERIAL**

**Table S1. Baseline Characteristics based on the persistence or discontinuation of dabigatran vs. warfarin.**

| Characteristic |  | Dabigatran | |  |  | Warfarin | |  |
| --- | --- | --- | --- | --- | --- | --- | --- | --- |
|  | Overall  N=459 | Persistence  Dabigatran  N=290 | Discontinuation  Dabigatran  N=169 | P value | Overall  N=6691 | Persistence  Warfarin  N=5532 | Discontinuation  Warfarin  N=1159 | P value |
|  |  |  |  |  |  |  |  |  |
| Age (years) | 71 (64-79) | 72 (64-78) | 71 (63-79) | .9 | 75 (68-82) | 76 (69-82) | 73 (64-80) | <.0001 |
| Male | 58 | 55 | 63 | .1 | 57 | 57 | 58 | .5 |
| Medical History |  |  |  |  |  |  |  |  |
| Hypertension | 82 | 83 | 80 | .4 | 85 | 86 | 82 | .005 |
| Hyperlipidemia | 71 | 70 | 71 | .9 | 74 | 75 | 70 | .003 |
| Smoking | 5.0 | 3.1 | 8.3 | .045 | 5.4 | 5.4 | 5.5 | .2 |
| Thyroid Disease |  |  |  |  |  |  |  |  |
| Hyperthyroidism | 1.3 | 1.0 | 1.8 | .5 | 2.3 | 2.3 | 2.0 | .5 |
| Hypothyroidism | 19 | 21 | 14 | .04 | 21 | 21 | 20 | .8 |
| Obstructive Sleep Apnea | 21 | 20 | 22 | .7 | 19 | 19 | 20 | .3 |
| Diabetes Mellitus | 27 | 28 | 26 | .6 | 30 | 31 | 28 | .1 |
| Prior CVA/TIA | 11 | 13 | 8.0 | .08 | 16 | 17 | 13 | .007 |
| CAD | 34 | 33 | 36 | .5 | 36 | 38 | 31 | <.0001 |
| Heart Failure  (NYHA Class III/IV) | 4.6 | 4.5 | 4.7 | .6 | 7.4 | 8 | 6 | .0006 |
| Sick Sinus Syndrome | 13 | 13 | 12 | .7 | 19 | 19 | 15 | .0008 |
| Sig. Valvular Disease | 17 | 16 | 20 | .3 | 28 | 29 | 22 | <.0001 |
| Cancer | 22 | 26 | 17 | .04 | 24 | 24 | 23 | .3 |
| Blood Pressure (diastolic) | 72 (68-80) | 72 (68-80) | 74 (67-80) | .8 | 72 (66-80) | 72 (66-80) | 72 (68-80) | .0007 |
| Blood Pressure (systolic) | 124 (115-136) | 124 (115-136) | 124 (116-135) | .8 | 126 (116-138) | 126 (116-138) | 126 (116-138) | .4 |
| BMI (kg/m^2^) | 30 (26-34) | 30 (26-36) | 29 (26-32) | .03 | 29 (26-34) | 29 (26-34)) | 29 (26-34) | .7 |
| Creatinine Clearance | 78 (58-106) | 79 (59-107) | 78 (57-102) | .5 | 69 (50-95) | 68 (50-93) | 75 (52-101) | <.0001 |
| LVEF (Normal) (%) | 76 | 76 | 77 | .7 | 69 | 69 | 71 | .01 |
| Type of AF |  |  |  | .3 |  |  |  | <.0001 |
| First Detected | 12 | 11 | 14 |  | 3.2 | 2.5 | 6.5 |  |
| Paroxysmal | 50 | 49 | 51 |  | 46 | 45 | 51 |  |
| Persistent | 21 | 21 | 22 |  | 18 | 17 | 18 |  |
| Permanent | 17 | 19 | 13 |  | 33 | 35 | 24 |  |
| Current AF Management Strategy |  |  |  | .6 |  |  |  | <.0001 |
| Rate Control | 57 | 42 | 56 |  | 72 | 73 | 63 |  |
| Rhythm Control | 43 | 41 | 44 |  | 28 | 26 | 37 |  |
| Prior AAD Treatment | 50 | 42 | 53 | .3 | 45 | 44 | 50 | <.0001 |
| Catheter Ablation of AF | 10 | 8 | 13 | .08 | 5.2 | 4.4 | 8.8 | <.0001 |
| Prior Warfarin Use | 63 | 67 | 57 | .04 | 94 | 95 | 92 | .001 |
| CHA_2_DS_2_VASC Risk Score | 4 (2-5) | 4 (2-5) | 3 (2-5) | .3 | 4 (3-5) | 4 (3-5) | 4 (2-5) | <.0001 |
| EHRA Score (Severe) | 20 | 18 | 24 | .2 | 14 | 13 | 16 | .004 |
| AFEQT Score at 1 year | 93 (75-95) | 86 (75-94) | 94 (84-99) | .1 | 83 (70-94) | 82 (69-94) | 88 (74-97) | .0012 |
| AF Symptoms |  |  |  |  |  |  |  |  |
| Palpitations | 40 | 37 | 44 | .1 | 31 | 29 | 36 | <.0001 |
| Dyspnea on Exertion | 31 | 26 | 38 | .009 | 28 | 28 | 29 | .4 |
| Fatigue | 32 | 26 | 41 | .0007 | 26 | 25 | 28 | .1 |
| Chest Tightness/  Discomfort | 13 | 12 | 13 | .9 | 8.4 | 8.0 | 10.4 | .006 |

Values presented as percentages or median (interquartile range). AFEQT, Atrial Fibrillation Effect on Quality of Life; AAD; antiarrythmic drug; BMI, body mass index (kg/m^2^ ); Creatinine clearance (ml/ min per 1.73m^2^) calculated by Cockcroft-Gault formula; EHRA, European Heart Rhythm Association; LVEF, left ventricular ejection fraction; NYHA, New York Heart Association.

**Table S2. List of covariates for propensity score**

**Demographics**

1. Age
2. Race- African American/Hispanic/White/Others
3. Gender- Male/Female
4. Level of Education- Some School/High School Graduate/College Graduate/Post Graduate
5. Geographic Region

**Medical History**

1. Smoking – Current/Recent or Former/Non-smoker
2. Hypertension – Yes/No
3. Diabetes – Yes/No
4. Obstructive Sleep Apnea – Yes/No
5. Cognitive Impairment/Dementia – Yes/No
6. History of alcohol use – Yes/No

**Cardiovascular History**

1. Stroke or TIA – Yes/No
2. Congestive Heart Failure (CHF) – No CHF/NYHA Class I/NYHA Class II/NYHA Class III or NYHA Class IV
3. Implanted Device – Pacemaker/ICD/BIV/BIV ICD

**Coronary Artery Disease History**

1. Prior MI – Yes/No

**Echocardiographic Assessment**

1. Evidence of Left Ventricular Hypertrophy

**Vital Signs & AF status**

1. Heart Rate, bpm
2. Systolic Blood Pressure, mmHG
3. Body Mass Index, kg/m2 (For imputation purpose, we will impute individual components which are weight, height)
4. Most Recent 12 Lead EKG – Sinus Rhythm

**Laboratory Data**

1. Hemotocrit, % (fill in from Hemoglobin by Hemoglobin*3 if missing)
2. Glomerular Filtration Rate, eGFR

**Atrial Fibrillation Diagnosis**

1. Type of AF – First Detected or New Onset/Paroxysmal AF/Persistent AF /Permanent AF
2. Prior Cardioversions – Yes/No
3. Catheter Ablation of AF – Yes/No
4. AV Node or HIS Bundle Ablation – Yes/No
5. Surgical Maze/Hybrid Maze

**Pharmacotherapy**

1. History of warfarin use

**Risk Scores**

1. CHA_2_DS_2_-VASc Score

**Table S3. Subgroup analyses for unadjusted and adjusted persistence rate of warfarin and dabigatran**

- Subgroup analyses for unadjusted persistence rate of warfarin and NOACs
- Age > 75 years

| *Medication* | *month* | *Persistence (%)* | *Lower Limit (%)* | *Upper Limit (%)* | *P-value* |
| --- | --- | --- | --- | --- | --- |
| Warfarin | 6 Months | 91.69 | 90.74 | 92.64 | <.0001 |
| NOAC | 6 Months | 70.06 | 62.82 | 77.31 | . |
| Warfarin | 1 Year | 85.66 | 84.48 | 86.85 | <.0001 |
| NOAC | 1 Year | 61.45 | 53.96 | 68.93 | . |

- Women

| *Medication* | *month* | *Persistence (%)* | *Lower Limit (%)* | *Upper Limit (%)* | *P-value* |
| --- | --- | --- | --- | --- | --- |
| Warfarin | 6 Months | 89.74 | 88.60 | 90.87 | <.0001 |
| NOAC | 6 Months | 79.78 | 73.91 | 85.66 | . |
| Warfarin | 1 Year | 83.02 | 81.64 | 84.40 | <.0001 |
| NOAC | 1 Year | 67.53 | 60.88 | 74.17 | . |

- CrCl < 50

| *Medication* | *month* | *Persistence (%)* | *Lower Limit (%)* | *Upper Limit (%)* | *P-value* |
| --- | --- | --- | --- | --- | --- |
| Warfarin | 6 Months | 89.51 | 88.14 | 90.87 | <.0001 |
| NOAC | 6 Months | 73.42 | 63.46 | 83.38 | . |
| Warfarin | 1 Year | 82.97 | 81.33 | 84.61 | <.0001 |
| NOAC | 1 Year | 58.33 | 47.57 | 69.10 | . |

- Single antiplatelet therapy, which was defined as getting only one medication among aspirin, clopidogrel, prasugrel, and ticagrelor

| *Medication* | *month* | *Persistence (%)* | *Lower Limit (%)* | *Upper Limit (%)* | *P-value* |
| --- | --- | --- | --- | --- | --- |
| Warfarin | 6 Months | 88.75 | 87.46 | 90.04 | <.0001 |
| NOAC | 6 Months | 70.75 | 63.31 | 78.19 | . |
| Warfarin | 1 Year | 81.78 | 80.23 | 83.33 | <.0001 |
| NOAC | 1 Year | 54.84 | 46.92 | 62.76 | . |

- Single or dual antiplatelet therapy, which was defined as getting at least one medication among aspirin, clopidogrel, prasugrel, and ticagrelor

| *Medication* | *month* | *Persistence (%)* | *Lower Limit (%)* | *Upper Limit (%)* | *P-value* |
| --- | --- | --- | --- | --- | --- |
| Warfarin | 6 Months | 88.25 | 86.98 | 89.52 | <.0001 |
| NOAC | 6 Months | 70.67 | 63.30 | 78.04 | . |
| Warfarin | 1 Year | 81.32 | 79.80 | 82.83 | <.0001 |
| NOAC | 1 Year | 55.06 | 47.22 | 62.90 | . |

- Subgroup analyses for adjusted persistence rate of warfarin and NOACs
- Age > 75 years

| *Medication* | *month* | *Persistence (%)* | *Lower Limit (%)* | *Upper Limit (%)* | *P-value* |
| --- | --- | --- | --- | --- | --- |
| Warfarin | 6 Months | 91.43% | 89.83% | 93.02% | <.0001 |
| NOAC | 6 Months | 70.67% | 61.40% | 79.94% | . |
| Warfarin | 1 Year | 85.15% | 82.87% | 87.43% | <.0001 |
| NOAC | 1 Year | 57.78% | 47.11% | 68.46% | . |

- Women

| *Medication* | *month* | *Persistence (%)* | *Lower Limit (%)* | *Upper Limit (%)* | *P-value* |
| --- | --- | --- | --- | --- | --- |
| Warfarin | 6 Months | 89.00% | 87.22% | 90.78% | 0.0011 |
| NOAC | 6 Months | 77.58% | 69.11% | 86.05% | . |
| Warfarin | 1 Year | 81.92% | 79.71% | 84.13% | 0.0003 |
| NOAC | 1 Year | 67.28% | 58.01% | 76.55% | . |

- CrCl < 50

| *Medication* | *month* | *Persistence (%)* | *Lower Limit (%)* | *Upper Limit (%)* | *P-value* |
| --- | --- | --- | --- | --- | --- |
| Warfarin | 6 Months | 89.80% | 87.32% | 92.28% | <.0001 |
| NOAC | 6 Months | 62.91% | 44.88% | 80.94% | . |
| Warfarin | 1 Year | 83.23% | 80.36% | 86.10% | <.0001 |
| NOAC | 1 Year | 45.63% | 26.93% | 64.33% | . |

- Single antiplatelet therapy, which was defined as getting only one medication among aspirin, clopidogrel, prasugrel, and ticagrelor

| *Medication* | *month* | *Persistence (%)* | *Lower Limit (%)* | *Upper Limit (%)* | *P-value* |
| --- | --- | --- | --- | --- | --- |
| Warfarin | 6 Months | 87.93% | 85.41% | 90.45% | 0.0042 |
| NOAC | 6 Months | 74.37% | 64.18% | 84.55% | . |
| Warfarin | 1 Year | 80.57% | 77.48% | 83.67% | <.0001 |
| NOAC | 1 Year | 56.92% | 45.18% | 68.66% | . |

- Single or dual antiplatelet therapy, which was defined as getting at least one medication among aspirin, clopidogrel, prasugrel, and ticagrelor

| *Medication* | *month* | *Persistence (%)* | *Lower Limit (%)* | *Upper Limit (%)* | *P-value* |
| --- | --- | --- | --- | --- | --- |
| Warfarin | 6 Months | 87.58% | 85.10% | 90.06% | 0.0044 |
| NOAC | 6 Months | 74.36% | 64.56% | 84.15% | . |
| Warfarin | 1 Year | 80.15% | 77.14% | 83.16% | <.0001 |
| NOAC | 1 Year | 57.50% | 46.23% | 68.77% | . |
